# Increased Expression of TLR10 in B Cell Subsets Correlates with Disease Activity in Rheumatoid Arthritis

**DOI:** 10.1155/2018/9372436

**Published:** 2018-12-27

**Authors:** Ying Zhang, Rong Cao, Haijian Ying, Juping Du, Shuaishuai Chen, Na Wang, Bo Shen

**Affiliations:** ^1^School of Laboratory Medicine and Life Sciences, Wenzhou Medical University, Wenzhou, Zhejiang, China; ^2^Department of Clinical Laboratory, Taizhou Hospital of Zhejiang Province, Taizhou Enze Medical Center (Group), Taizhou, Zhejiang, China

## Abstract

Toll-like receptor (TLR) 10, mainly expressed on B cells, has emerged as a modulatory receptor in inflammation. Nonetheless, the clinical significance of TLR10 in rheumatoid arthritis (RA) remains unclear. In this study, we explored the expression of TLR10 in B cells and B cell subsets in RA subjects and healthy controls (HCs) and determined its relevance to disease activity and inflammatory biomarkers. TLR10 levels in B cells and B cell subsets (CD19^+^CD27^+^, CD19^+^CD27^−^, CD27^+^IgD^−^, CD27^+^IgD^+^, CD27^−^IgD^+^, D27^−^IgD^−^, CD19^+^CD5^+^, and CD19^+^CD5^−^) and inflammatory biomarker concentrations in peripheral blood (PB) obtained from RA subjects and HCs were detected by flow cytometry and enzyme-linked immunosorbent assay (ELISA), respectively. The correlations of TLR10 expression with disease activity and inflammatory biomarkers were then analysed. Similar levels of TLR10 in all CD19^+^ B cells were observed in the RA subjects and HCs. Compared to that in the HCs, TLR10 was elevated significantly in the CD19^+^CD27^−^IgD^−^ and CD19^+^CD5^+^ subsets in the RA subjects. In addition, almost all subsets expressing TLR10 were increased with disease activity. The present study reveals that enhanced TLR10 in B cell subsets is positively correlated with disease activity in RA subjects.

## 1. Introduction

Rheumatoid arthritis (RA) is a systemic autoimmune disease characterized by an immune system disturbance and chronic inflammation of the synovium, which leads to progressive joint destruction [1]. The specific mechanisms underlying disease development and progression remain unclear, despite the substantial amount of relevant literature. Clinical treatments mainly involve drugs to control disease activity and relieve symptoms.

In recent years, B cells have been proven to play a prominent role in RA. These cells are involved from the very beginning of their cycle to the secretion of autoantibodies, the presentation of antigens to activate T cells, and the secretion of proinflammatory cytokines [2]. Recently, the effectiveness of the anti-CD20 agent rituximab has been explored in RA [3]. Furthermore, the identification of different types of regulatory B cells that secrete anti-inflammatory cytokines and modulate tolerance [4, 5] indicates that B cells have pleiotropic effects in RA.

Toll-like receptors (TLRs) recognize a broad spectrum of pathogen-associated molecular patterns (PAMPs), which are induced by invading microorganisms [6] and damage-associated molecular patterns (DAMPs), such as endogenous nucleic acids [7, 8]. TLR induction leads to signalling cascades that play critical roles in innate immune and inflammatory responses. TLR10 is the most recently identified TLR [9] and is mainly expressed on B cells [10–13]. Similar to other TLRs, TLR10 is a transmembrane receptor composed of extracellular leucine-rich repeat-recognizing domains and an intracellular toll/IL-1 receptor homology (TIR) signalling domain [9]. Although the ligand(s) and downstream signalling pathways of TLR10 remain unknown, this receptor has been reported to be an immunomodulatory receptor with inhibitory properties [13–16], which distinguishes TLR10 from other TLRs.

In humans, genetic polymorphisms in TLR10 have been associated with autoimmune and infectious diseases and cancers, including Crohn's disease [17], thyroid disease [18], complicated skin and skin structure infections [19], tuberculosis [20], nasopharyngeal cancer [21], and non-Hodgkin's lymphoma [22]. In Korean populations or Caucasian European populations, no association was identified between two specific TLR10 variants (rs4129009 [23] and rs11466657 [24]) and RA susceptibility. However, the study of Torices et al. showed that rs11466657 is closely related to RA disease severity, and infliximab treatment is ineffective in patients carrying these variants [24].

The currently available data present only a flawed understanding about the relationship between TLR10 and RA. Considering the unique expression of TLR10 on B cells and the immunomodulatory properties of this receptor, in this study, we investigated TLR10 expression in B cell subsets in the peripheral blood (PB) obtained from RA subjects and analysed correlations of TLR10 expression with disease activity and inflammatory biomarkers.

## 2. Materials and Methods

### 2.1. Study Subjects

The present study received approval from the Medical Ethics Committee of Taizhou Hospital of Zhejiang Province, and informed consent was obtained from each subject.

From February 2018 to April 2018, 77 patients diagnosed with RA based on the 2010 American College of Rheumatology (ACR)/European League Against Rheumatism (EULAR) criteria at Taizhou Hospital, Zhejiang Province, China, were consecutively recruited. Subjects suffering from other autoimmune or inflammatory diseases, severe renal or liver disease, or cancer were excluded. The disease activity of each subject was determined according to the Disease Activity Score of 28 joints (DAS28). All the RA subjects were divided into three groups: a high-activity (HA) group (DAS28 ≥ 5.1), a moderate-activity (MA) group (3.2 ≤ DAS28 < 5.1), and a low-activity (LA) group (DAS28 < 3.2). Laboratory tests performed on each patient included rheumatoid factor (RF), anticyclic citrullinated peptide antibody (anti-CCP), C-reactive protein (CRP), and erythrocyte sedimentation rate (ESR).

In addition, 30 age- and sex-matched healthy controls (HCs) were chosen for comparison.

### 2.2. Blood Samples and Laboratory Testing

The serum samples used to detect the levels of inflammation-related factors were stored at −80°C. The levels of clinical laboratory indicators (e.g., ESR, CRP, anti-CCP, and RF) were determined using standard clinical laboratory protocols in the hospital.

### 2.3. Flow Cytometry Analysis

**Figure 1:**
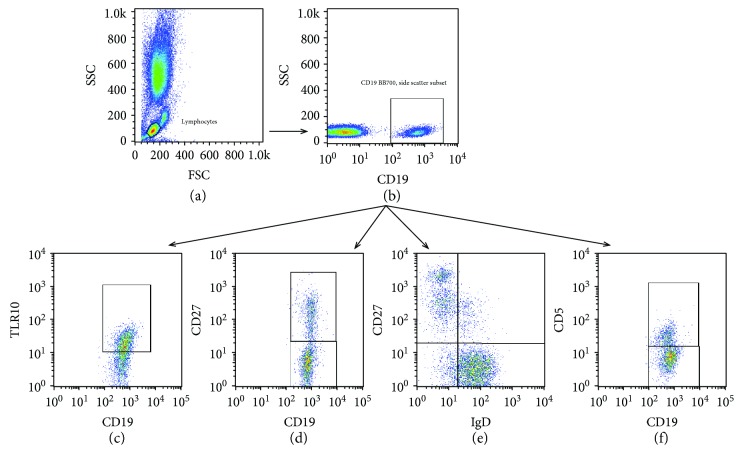
B cells, B cell subsets, and TLR10 gating strategy: (a) selecting lymphocytes based on side-scatter and forward-scatter; (b) selecting CD19^+^ B cells from lymphocytes; (c) analysing TLR10 in CD19^+^ B cells; (d-f) dividing CD19^+^ B cells into several subsets (CD19^+^CD27^+^, CD19^+^CD27^−^, CD19^+^CD27^+^IgD^−^, CD19^+^CD27^+^IgD^+^, CD19^+^CD27^−^IgD^+^, CD19^+^CD27^−^IgD^−^, CD19^+^CD5^+^, and CD19^+^CD5^−^) for further analysis of the TLR10 expression.

The immunophenotyping of B cells was performed in the PB samples (50 *μ*l) using the following fluorochrome-labelled antibodies: CD19-BB700, CD27-APC, IgD-FITC, CD5-FITC (all BD Biosciences), and TLR10-PE (BioLegend). Fresh whole blood was stained with antibodies for 20 minutes at room temperature (RT) in the dark, lysed with 500 *μ*l of lysis buffer (BD) for 15 minutes under the same conditions, washed twice with PBS, resuspended, and analysed by flow cytometry. Approximately 100,000 events were collected per sample. The data were collected with a FACSCalibur (BD Bioscience) and analysed using the FlowJo software version 10.0.  Figure 1 shows the results for CD19^+^ B cells and the subset gating strategy used in these experiments.

### 2.4. ELISA Measurements

Serum IL-1Ra, IL-1*β*, and IL-10 concentrations were determined in 77 RA subjects and 30 HCs using commercially available ELISA kits (IL-1Ra and IL-1*β* ELISA kit from Elabscience; IL-10 ELISA kit from Multisciences) according to the manufacturers' instructions.

### 2.5. Statistical Analysis

The results are expressed as the means ± standard error (SE) and medians (interquartile range). Statistical comparisons were performed by Student's *t*-tests and Mann–Whitney *U* tests. Differences among the three groups were determined by the Kruskal–Wallis *H* nonparametric test. Correlation analyses between two parameters were performed by Spearman's correlation method. All statistical analyses were performed using the SPSS software version 20 (SPSS Inc., Chicago, Illinois, USA). A *P* value < 0.05 was considered statistically significant.

## 3. Results

### 3.1. Characteristics of the Study Population

We enrolled 77 RA patients (19 LA patients, 29 MA patients, and 29 HA patients) and 30 HCs. Their detailed clinical characteristics are provided in Table 1. The average age of each group was 51.6 ± 7.0, 54.2 ± 8.8, 51.2 ± 7.8, 55.8 ± 8.5, and 54.4 ± 9.4, respectively. There were similar proportions of females in each group (86.7%, 85.7%, 84.2%, 86.2%, and 86.2%, respectively). The average age and gender were not significantly different among the groups.

### 3.2. The Percentage of TLR10-Expressing CD19^+^ B Cells in RA Subjects Is Associated with Disease Activity

**Figure 2:**
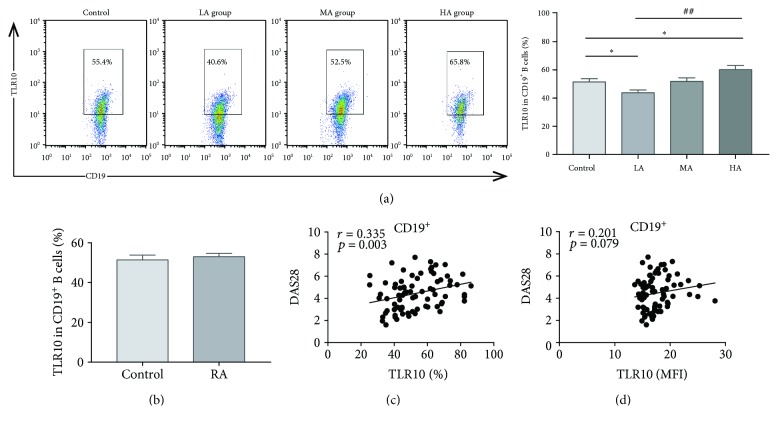
TLR10 expression in CD19^+^ B cells and its association with the DAS28. (a) A representative dot plot is presented for each group. (b) The percentage of cells expressing TLR10 (%TLR10^+^) in CD19^+^ B cells in the RA subjects and HCs. (c) The correlation between the %TLR10^+^ expression in CD19^+^ B cells and the DAS28. (d) The correlation between TLR10 MFI in CD19^+^ B cells and the DAS28. Differences between individual groups were analysed by the Mann–Whitney test and are described as ∗ (^∗^ *P* < 0.05); differences among the three groups of RA subjects were analysed by the Kruskal–Wallis test and are described as # (^##^ *P* < 0.01).

We studied the expression of TLR10 in the total CD19^+^ B cells obtained from RA subjects and HCs. Similar levels of TLR10 in all the CD19^+^ B cells were observed between the RA subjects and HCs regardless of whether they were analysed as a percentage or as the mean fluorescence intensity (MFI) (Table 2 and Figure 2(b)). However, in the RA subjects, the percentage of TLR10-expressing CD19^+^ B cells significantly increased with disease activity (*P* = 0.001) (Table 2 and Figure 2(a)). Compared with the HCs, the HA group expressed higher TLR10 levels in CD19^+^ B cells (60.2 ± 2.9% vs. 51.4 ± 2.4%; *P* = 0.024), while the LA group expressed lower levels of TLR10 (43.8 ± 2.0% vs. 51.4 ± 2.4%; *P* = 0.017) (Figure 2(a)). Furthermore, the percentage of CD19^+^TLR10^+^ cells in the RA subjects was positively related to the DAS28 (*r* = 0.335, *P* = 0.003) (Figure 2(c)), but no correlations were found for RF, anti-CCP, CRP, or ESR.

To evaluate inflammation-related factors, we determined the serum IL-1Ra, IL-1*β*, and IL-10 concentrations in the RA subjects and HCs (Table S1). Notably, in the RA subjects, the percentage and MFI of the TLR10-expressing CD19^+^ B cells were positively correlated with the IL-1*β* concentrations (*r* = 0.259, *P* = 0.023; *r* = 0.246, *P* = 0.031, respectively) (Figure S1).

### 3.3. TLR10 Is Mainly Expressed in the CD19^+^CD27^+^ Subset in the RA Subjects and HCs

**Figure 3:**
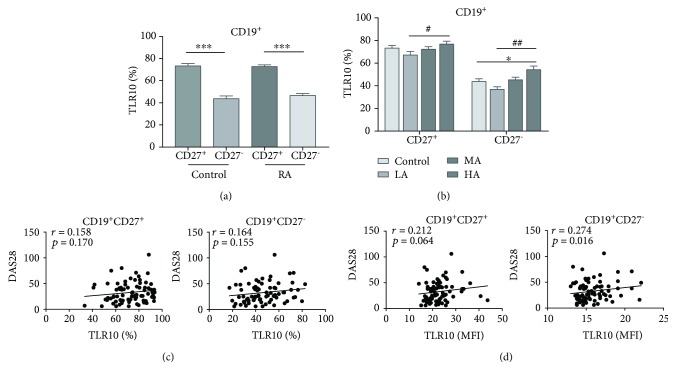
TLR10 expression in CD19^+^ B cell subsets (defined using CD27) and its association with the DAS28. (a) The percentage of cells expressing TLR10 (%TLR10^+^) in each subset in the RA subjects and HCs. (b) The percentage of cells expressing TLR10 (%TLR10^+^) in each group of each subset. (c) The correlation between %TLR10^+^ expression in each subset and the DAS28. (d) The correlation between the TLR10 MFI in each subset and the DAS28. Differences between individual groups were analysed by the Mann–Whitney test and are described as ∗ (^∗^ *P* < 0.05 and ^∗∗∗^ *P* < 0.001); differences among the three groups of RA subjects were analysed by the Kruskal–Wallis test and are described as # (^#^ *P* < 0.05 and ^##^ *P* < 0.01).

Because CD27 is a marker of B cell maturation [25], we analysed TLR10 expression in the CD19^+^CD27^+^ and CD19^+^CD27^−^ subsets obtained from both the RA subjects and HCs. The results showed that TLR10 was mainly expressed in the CD19^+^CD27^+^ subset in both the RA subjects and HCs (72.7 ± 1.6% vs. 46.5 ± 1.8% and 73.2 ± 2.2% vs. 43.7 ± 2.4%; *P* ≤ 0.001 and *P* ≤ 0.001, respectively) and that TLR10 levels were similar between the RA subjects and HCs in both the CD19^+^CD27^+^ and the CD19^+^CD27^−^ subsets (*P* = 0.983 and *P* = 0.385, respectively) (Table 2 and Figure 3(a)). In the RA subjects, TLR10 expression significantly increased with disease activity in both the CD19^+^CD27^+^ subset and the CD19^+^CD27^−^ subset (*P* = 0.038 and *P* = 0.001, respectively) (Table 2 and Figure 3(b)). Interestingly, the percentage of CD19^+^CD27^−^TLR10^+^ cells was dramatically upregulated in the HA group compared with the HCs (54.2 ± 3.2% vs. 43.7 ± 2.4%, *P* = 0.019) (Figure 3(b)). Moreover, in the CD19^+^CD27^+^ subset, the percentage and MFI of TLR10 were positively associated with the ESR (*r* = 0.297, *P* = 0.009 and *r* = 0.310, *P* = 0.006, respectively) (Figure S2), whereas in the CD19^+^CD27^−^ subset, the MFI of TLR10 was positively related to the DAS28 (*r* = 0.274, *P* = 0.016) (Figure 3(d)).

### 3.4. The Percentage of CD27^−^IgD^−^TLR10^+^ Cells Is Significantly Higher in the RA Subjects

To determine whether TLR10 is differentially expressed among B cell subsets, we further subdivided B cells into the four canonical B cell subsets: naive cells (CD27^−^IgD^+^), preswitched memory cells (CD27^+^IgD^+^), switched memory cells (CD27^+^IgD^−^), and double-negative (DN) cells (CD27^−^IgD^−^) [26].

**Figure 4:**
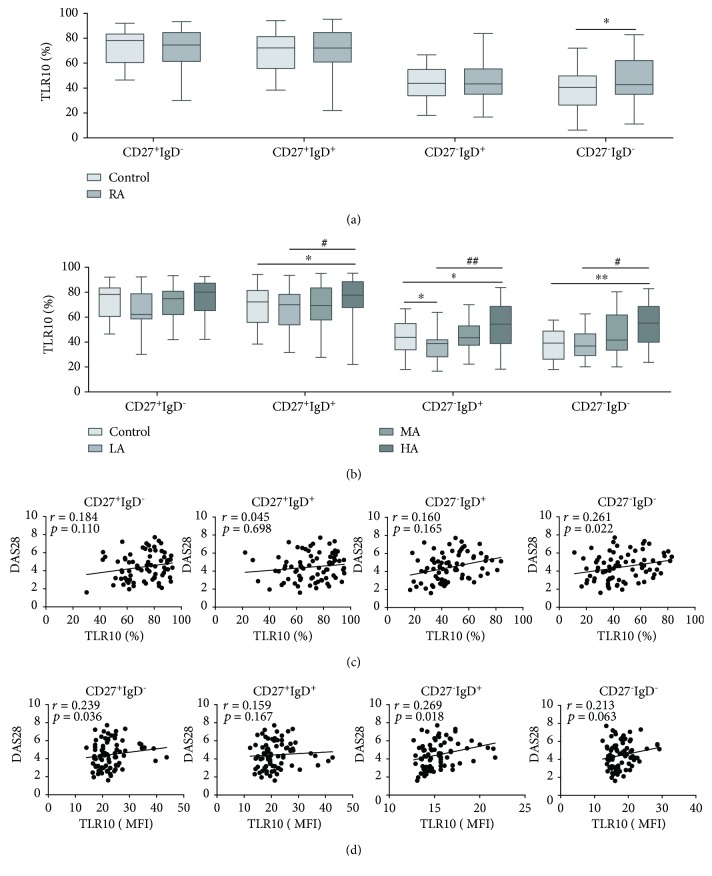
TLR10 expression in CD19^+^ B cell subsets (defined using IgD/CD27) and its association with the DAS28. (a) The percentage of cells expressing TLR10 (%TLR10^+^) in each subset in the RA subjects and HCs. (b) The percentage of cells expressing TLR10 (%TLR10^+^) in each group of each subset. (c) The correlation between %TLR10^+^ expression in each subset and the DAS28. (d) The correlation between the TLR10 MFI in each subset and the DAS28. The data are presented as the median (interquartile range (IQR)) using whisker-box plots, with the boxes representing the IQR and the whiskers representing the min/max values. Differences between individual groups were analysed by the Mann–Whitney test and are described as ∗ (^∗^ *P* < 0.05 and ^∗∗^ *P* < 0.01); differences among the three groups of RA subjects were analysed by the Kruskal–Wallis test and are described as # (^#^ *P* < 0.05 and ^##^ *P* < 0.01).

In the CD27^−^IgD^−^ subset, the TLR10 expression was remarkably enhanced in the RA subjects compared to the HCs (*P* = 0.047) (Table 2 and Figure 4(a)), especially in the RA subjects with severe disease activity (Figure 4(b)). Elevated TLR10 levels with increased disease activity were found in this subset (*P* = 0.016) (Table 2 and Figure 4(b)). The relative analysis indicated that the percentage of TLR10 was in direct proportion to the DAS28 (*r* = 0.261, *P* = 0.022) (Figure 4(c)) and the ESR (*r* = 0.254, *P* = 0.026) (Figure S3(a)); the MFI of TLR10 was positively correlated with the ESR (*r* = 0.308, *P* = 0.006) (Figure S3(b)).

In the remaining three subsets, the TLR10 expression was not remarkably different between the RA subjects and HCs (Table 2 and Figure 4(a)). However, among the three disease activity groups, there was an increasing trend for the TLR10 levels in the RA subjects, and this tendency reached significance in all but the CD27^+^IgD^−^ subset (*P* = 0.054, *P* = 0.026, and *P* = 0.001, respectively) (Table 2 and Figure 4(b)). Moreover, in the CD27^+^IgD^−^ and CD27^−^IgD^+^ subsets, the MFI of TLR10 correlated positively with the DAS28 (*r* = 0.239 and *P* = 0.036 and *r* = 0.269 and *P* = 0.018, respectively) (Figure 4(d)). In the CD27^+^IgD^−^ subset, the percentage and MFI of TLR10 correlated positively with the ESR (*r* = 0.304 and *P* = 0.007 and *r* = 0.244 and *P* = 0.032, respectively) (Figure S3).

### 3.5. The Percentage of CD19^+^CD5^+^TLR10^+^ Cells Was Significantly Higher in the RA Subjects

**Figure 5:**
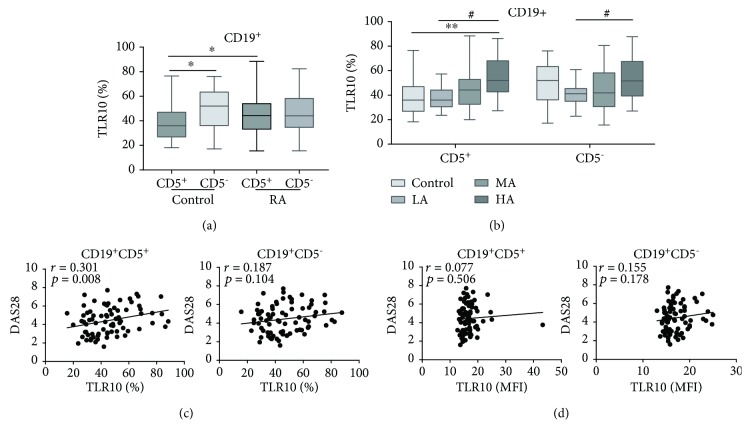
TLR10 expression in CD19^+^ B cell subsets (defined using CD5) and its association with the DAS28. (a) The percentage of cells expressing TLR10 (%TLR10+) in each subset of the RA subjects and HCs. (b) The percentage of cells expressing TLR10 (%TLR10^+^) in each group of each subset. (c) The correlation between %TLR10^+^ expression in each subset and the DAS28. (d) The correlation between the TLR10 MFI in each subset and the DAS28. The data are presented as the median (interquartile range (IQR)) using whisker-box plots, with boxes representing the IQR and the whiskers representing the min/max values. Differences between individual groups were analysed by the Mann–Whitney test and are described as ∗ (^∗∗^ *P* < 0.01); differences among the three groups of RA subjects were analysed by the Kruskal–Wallis test and are described as # (^#^ *P* < 0.05).

An analysis of the CD5 expression showed that B cells could be further divided into the CD19^+^CD5^+^ subset and the CD19^+^CD5^−^ subset. In the HCs, the proportion of CD19^+^CD5^+^TLR10^+^ cells (34.6 (25.7-46.8) %) was significantly lower than that of CD19^+^CD5^−^TLR10^+^ cells (52.1 (36.2-63.4) %) (*P* = 0.013) (Figure 5(a)). However, in the RA subjects, the percentages of the two subsets were not significantly different because there was an increase in the percentage of the CD19^+^CD5^+^TLR10^+^ B cells and a decrease in the percentage of CD19^+^CD5^−^TLR10^+^ B cells. In the CD19^+^CD5^+^ subset, the percentage with TLR10 increased markedly in the RA subjects compared to the HCs (*P* = 0.024) (Table 2 and Figure 5(a)). Among the three disease activity groups, an elevation of TLR10 with increased disease activity was observed in this subset, regardless of whether the percentages or MFI was assessed (*P* = 0.002 or *P* = 0.034, respectively) (Table 2 and Figure 5(b)). Importantly, the percentage of CD19^+^CD5^+^TLR10^+^ B cells was positively related to the DAS28 (*r* = 0.301, *P* = 0.008) (Figure 5(c)).

Although the decrease in the percentage of CD19^+^CD5^−^TLR10^+^ B cells observed in the RA subjects was not prominent compared to that in the HCs, the TLR10 levels greatly increased with disease activity in the RA subjects (*P* = 0.033) (Table 2 and Figure 5(b)).

## 4. Discussion

The present results reveal that the total level of TLR10 expression in all CD19^+^ B cells was not significantly different between the RA subjects and HCs, while TLR10 was expressed at different levels among different B cell subsets and was mainly expressed in the CD27^+^ and CD5^−^ subsets in both the RA subjects and HCs. The RA subjects showed a marked increase in TLR10 expression in the CD27^−^IgD^−^ and CD5^+^ subsets, whereas slightly decreased expression was observed in the rest of the subsets, compared to the HCs. Moreover, in this paper, we also identified correlations of the TLR10 expression with disease activity and inflammatory biomarkers in B cells and B cell subsets.

The expression of TLR10 in total CD19^+^ B cells was associated with the DAS28 and IL-1*β* levels in the RA subjects, indicating that an inflammatory state was present in the body and that TLR10 acts as an inflammation-associated protein in this inflammatory disease. This finding is consistent with previous studies reporting that TLR10 variants have no association with RA susceptibility in either a Korean population or a Caucasian European population but are highly associated with RA severity [23, 24].

In addition, TLR10 expression clearly increased with disease activity in almost all subsets. Bourke et al. reported that stimulating B cells with BCR and anti-CD40 antibodies or *Staphylococcus aureus* Cowan I bacteria (SAC) strongly induced TLR10 gene expression [11]. Other reports demonstrated that reactive oxygen species also enhanced TLR10 expression in THP-1 cells [27, 28]. These data indicate that inflammatory conditions can upregulate TLR10 expression. RA is a chronic inflammatory autoimmune disease associated with abnormal increases in inflammatory factors, such as IL-1*β* [29], which may stimulate B cells to induce TLR10 expression, especially in severe disease states. However, in this study, the TLR10 expression in almost all B cell subsets in the LA group of RA subjects was lower than that in the HCs. In this disease state, TLR10 expression may not have been upregulated by inflammatory stimulation.

Regarding the difference in the expression of TLR10 among the B cell subsets, TLR10 levels were significantly higher in the CD19^+^CD27^+^ subset than in the CD19^+^CD27^−^ subset in both the RA subjects and HCs. This finding is not entirely surprising because CD27 represents a critical marker that is activated in mature B cells [25], and TLR10 expression is upregulated during the maturation and activation process in B lymphocytes [11, 12]. These results suggest that TLR10 levels reflect the activation and differentiation states of cells.

Among the canonical B cell subsets, we showed that TLR10 levels were significantly higher in the CD27^−^IgD^−^ subset in the RA subjects than in the HCs, whereas these levels were slightly decreased in the rest of the subsets. We have no good explanation for these results, but we speculate that TLR10 functions as an inhibitory receptor, as evidenced by its association with the inhibition of proinflammatory factor production and B cell proliferation and differentiation [13] and the induction of trophoblast apoptosis [30]. Thus, there are at least two potential explanations for this result. On the one hand, considering the immunosuppressive properties of TLR10, CD27^+^ memory B cells and naive B cells may tend to be more inflammatory because these cells express less TLR10, which causes the proinflammatory effects of the cells to prevail and thereby promotes the development of RA. On the other hand, the increased expression of TLR10 observed in CD27^−^IgD^−^ cells shows that immune regulation is increased, which may allow TLR10 to exert immunosuppressive effects, in line with the balance between proinflammatory and immunosuppressive effects in the body. This finding may also explain why there was no significant difference in TLR10 expression in total CD19^+^ B cells between the RA subjects and HCs. However, TLR10 has an opposite function in infectious disease; this receptor localizes in the cytoplasm of monocyte-derived macrophages, THP-1 cells, and intestinal epithelial cells, where TLR10 can exert a proinflammatory effect because live pathogens can only replicate and interact with TLR10 in the cytoplasm [31, 32]. Indeed, the study about the TLR10 localization to endosomes and its ability to recognize dsRNA were both reported during our study [33]. Therefore, the role of TLR10 in that case is different from the role we studied on the cell surface.

This phenomenon was also corroborated by our findings in the CD5^+^ and CD5^−^ subsets. CD5^+^ B cells, also called B-1a cells, contribute to innate immunity and play a central role in immunoregulation, whereas CD5^−^ B cells consist of B-1b cells and B-2 cells, which participate in acquired immunity [34, 35]. According to this classification, in our study, TLR10 was expressed at significantly higher levels in the CD19^+^CD5^+^ subset in the RA subjects than in the HCs, while there was a small decrease in TLR10 expression in the CD19^+^CD5^−^ subset in the RA subjects, consistent with our above conclusion that B cell subsets with proinflammatory properties show decreased TLR10 expression and tend to be more inflammatory, while B cell subsets with immunomodulatory properties show increased TLR10 expression and act to inhibit inflammation.

There were several limitations to this research. (1) Our sample size was relatively small, though the results were significant. Further studies with a larger sample size are essential to verify our findings. (2) The present study was restricted to Chinese individuals. Therefore, more proof is required to confirm the validity of these findings in other races/ethnic populations. (3) Our study lacked imaging data to demonstrate the relationship between TLR10 and RA progression.

## 5. Conclusions

In conclusion, we found that levels of TLR10 were increased in CD27^−^IgD^−^ and CD19^+^CD5^+^ B cells in RA subjects and were positively correlated with disease activity, revealing that TLR10 as an immunosuppressive factor may play a critical role in the progression of RA. The results from the present study support further investigation of the possible molecular mechanisms of TLR10 in the pathogenesis of RA.

## Figures and Tables

**Table 1:** Clinical characteristics of the study subjects.

	Healthy control group (*n* = 30)	All RA group (*n* = 77)	Low-activity RA group (*n* = 19)	Moderate-activity RA group (*n* = 29)	High-activity RA group (*n* = 29)
Female (%)	26 (86.7)	66 (85.7)	16 (84.2)	25 (86.2)	25 (86.2)
Age (years)	51.6 ± 7.0	54.2 ± 8.8	51.2 ± 7.8	55.8 ± 8.5	54.4 ± 9.4
Disease duration (years)	—	5.0 (2-12)	3.5 (2-13)	7 (2.5-12.5)	4 (1.5-10)
RF (kU/l)^a^	—	65.1 (34.8-187)	63.1 (37.9-146.8)	62.1 (29.0-150)	93.5 (34.6-259)
RF-positive (%)^a^	—	62 (87.3)	15 (93.8)	24 (85.7)	23 (85.2)
Anti-CCP (U/ml)^b^	—	94.1 (31.7-532.3)	87.1 (22.4-536.1)	91.4 (49.2-501)	100.9 (34.8-574)
Anti-CCP-positive (%)^b^	—	67 (89.3)	16 (88.9)	26 (89.7)	25 (89.3)
CRP (mg/l)^c^	—	3.0 (0.9-9.9)	1.3 (0.8-3.6)	2.4 (0.9-9.3)	5.1 (1.2-17.3)
CRP-positive (%)^c^	—	20 (28.6)	1 (6.7)	8 (27.6)	11 (42.3)
ESR (mm/h)	—	27 (18-42)	19 (11-26)	33 (23-46)	38 (21.5-51.5)
DAS28-ESR	—	4.4 ± 1.6	2.6 ± 0.4	4.0 ± 1.0	5.9 ± 0.9
Medicine use	—				
NSAIDs	—	16 (20.8)	2 (10.5)	8 (27.6)	6 (20.7)
DMARDs	—	57 (74.0)	17 (89.5)	20 (69.0)	20 (69.0)
Methotrexate	—	47 (61.0)	15 (78.9)	15 (51.7)	17 (58.6)
Leflunomide	—	32 (41.6)	10 (52.6)	11 (37.9)	11 (37.9)
Sulfasalazine	—	8 (10.4)	2 (10.5)	5 (17.2)	1 (3.4)
Prednisolone	—	12 (15.6)	2 (10.5)	3 (10.3)	7 (24.1)
Chinese medicine	—	16 (20.8)	4 (21.1)	7 (24.1)	7 (24.1)
No treatment	—	7 (9.1)	1 (5.3)	2 (6.9)	4 (13.8)
Other^d^	—	4 (5.2)	1 (5.3)	2 (6.9)	1 (3.4)
Unknown^e^	—	4 (5.2)	0 (0)	1 (3.4)	3 (10.3)

The data are expressed as *n* (%), mean ± standard deviation (SD), or median (interquartile range, 25th-75th). DAS28-ESR (Disease Activity Score of 28 joints using ESR), NSAIDs (nonsteroidal anti-inflammatory drugs), and DMARDs (disease-modifying antirheumatic drugs). ^a^RF data were lacking in 6 subjects. Sixty-two out of 71 RA subjects were RF-positive. ^b^Anti-CCP data were lacking in 2 subjects. Sixty-seven out of 75 RA subjects were anti-CCP-positive. ^c^CRP data were lacking in 7 subjects. Twenty out of 70 RA subjects were CRP-positive. ^d^The patients defined as “other” included the following: those who were on their first visit to our hospital, were not on regular medication (one patient), and were taking other medicines that were not related to RA therapy (three patients). ^e^The patients defined as “unknown” included the following: those who were on their first visit to our hospital, could not tell which medication to use (two patients), and were only told of a few of their medicines (two patients).

**Table 2:** TLR10 expression in B cells and B cell subsets in the study subjects.

	Control (*n* = 30)	RA (*n* = 77)	*P* ^∗^	LA (*n* = 19)	MA (*n* = 29)	HA (*n* = 29)	*P* ^#^
CD19^+^							
%	51.4 ± 2.4	53.0 ± 1.7	0.612	43.8 ± 2.0	51.9 ± 2.5	60.2 ± 2.9	0.001
MFI	17.5 ± 0.4	17.5 ± 0.3	0.908	16.2 ± 0.2	17.1 ± 0.4	18.7 ± 0.6	0.03
CD19^+^CD27^+^							
%	73.3 ± 2.2	72.7 ± 1.6	0.829	67.1 ± 3.3	72.2 ± 7.4	76.8 ± 2.6	0.038
MFI	22.7 ± 1.0	22.8 ± 0.6	0.932	20.8 ± 0.7	21.8 ± 0.7	25.1 ± 1.3	0.093
CD19^+^CD27^−^							
%	43.7 ± 2.4	46.5 ± 1.8	0.385	36.8 ± 2.4	45.2 ± 2.3	54.2 ± 3.2	0.001
MFI	15.4 ± 0.3	15.9 ± 0.2	0.207	20.8 ± 0.7	15.6 ± 0.3	16.9 ± 0.5	≤0.001
CD19^+^CD27^+^IgD^−^							
%	78.3 (60.6-83.5)	74.5 (61.5-84.3)	0.914	62.1 (58.6-78.8)	74.8 (62.0-80.8)	80.1 (65.3-87.3)	0.054
FI	22.5 (19.0-25.1)	21.9 (19.5-25.0)	0.819	20.4 (18.4-22.9)	21.7 (19.2-25.2)	22.1 (20.2-31.1)	0.097
CD19^+^CD27^+^IgD^+^							
%	72.3 (55.8-81.4)	72.2 (61.0-84.6)	0.479	70.0 (53.9-78.3)	69.4 (57.8-83.5)	77.6 (67.8-88.5)	0.026
FI	18.8 (17.5-22.6)	20.6 (17.9-24.2)	0.276	18.3 (16.4-22.1)	19.8 (18.1-25.8)	21.9 (18.5-26.1)	0.101
CD19^+^CD27^−^IgD^+^							
%	43.8 (33.9-55.0)	43.4 (35.1-55.4)	0.737	38.9 (28.2-41.9)	43.5 (37.5-53.1)	54.4 (38.9-68.6)	0.001
FI	15.4 (14.1-16.4)	15.0 (13.9-16.4)	0.605	14.1 (13.8-14.6)	15.5 (14.0-15.9)	15.7 (14.6-18.1)	0.002
CD19^+^CD27^−^IgD^−^							
%	40.5 (26.4-49.8)	42.8 (35.0-62.2)	0.047	36.7 (26.1-46.4)	41.6 (33.5-61.8)	54.5 (39.0-67.9)	0.016
MFI	16.7 (15.0-18.3)	17.0 (15.3-20.4)	0.167	16.3 (15.3-17.2)	18.1 (15.1-20.7)	18.3 (15.7-21.7)	0.126
CD19^+^CD5^+^							
%	34.6 (25.7-46.8)	44.3 (33.2-54.0)	0.024	36.0 (30.5-44.2)	44.3 (32.6-52.8)	52.0 (42.7-68.0)	0.002
MFI	16.1 (14.1-17.0)	16.1 (14.6-17.5)	0.397	14.6 (14.2-16.8)	16.4 (15.2-17.3)	16.5 (15.0-18.6)	0.034
CD19^+^CD5^−^							
%	52.1 (36.2-63.4)	45.0 (34.9-59.0)	0.435	41.1 (35.0-45.4)	41.9 (30.7-58.2)	51.7 (39.4-67.6)	0.033
MFI	16.9 (15.5-19.0)	16.5 (15.3-18.5)	0.304	15.8 (15.0-17.2)	15.8 (15.0-19.0)	16.8 (15.8-18.9)	0.098

The data are shown as the means ± SE or median (interquartile range, 25th-75th). ∗ represents a comparison between the RA subjects and HCs based on the Mann–Whitney test. # represents a comparison among the three groups of RA subjects based on the Kruskal–Wallis test.

## Data Availability

The data used to support the findings of this study are available from the corresponding author upon request.

## Supplementary Materials

Supplementary Materials Table S1: serum concentration of cytokines in all participants. Figure S1: the correlation between %TLR10^+^ (a) and TLR10 MFI (b) in CD19^+^ B cells and IL-1*β* concentrations. Figure S2: the correlation between %TLR10^+^ (a) and TLR10 MFI (b) in CD19^+^CD27^+^ B cells and ESR. Figure S3: (a) the correlation between %TLR10^+^ in CD27^+^IgD^−^ B cells and ESR. (b) The correlation between TLR10 MFI in CD27^+^IgD^+^ B cells and ESR.
