# Erratum to “Increased Expression of TLR10 in B Cell Subsets Correlates with Disease Activity in Rheumatoid Arthritis”

**DOI:** 10.1155/2019/8419439

**Published:** 2019-03-10

**Authors:** Ying Zhang, Rong Cao, Haijian Ying, Juping Du, Shuaishuai Chen, Na Wang, Bo Shen

**Affiliations:** ^1^School of Laboratory Medicine and Life Sciences, Wenzhou Medical University, Wenzhou, Zhejiang, China; ^2^Department of Clinical Laboratory, Taizhou Hospital of Zhejiang Province, Taizhou Enze Medical Center (Group), Taizhou, Zhejiang, China

In the article titled “Increased Expression of TLR10 in B Cell Subsets Correlates with Disease Activity in Rheumatoid Arthritis” [1], the email address of the corresponding author Bo Shen should be corrected as “shenbkz@aliyun.com.” In addition, the format of Table 1 was incorrect. This occurred due to a production error. The corrected Table is shown below.

## Figures and Tables

**Table 1:** Clinical characteristics of the study subjects.

	Healthy control group (*n* = 30)	All RA group (*n* = 77)	Low active RA group (*n* = 19)	Moderate active RA group (*n* = 29)	High active RA group (*n* = 29)
Female (%)	26 (86.7)	66 (85.7)	16 (84.2)	25 (86.2)	25 (86.2)
Age (years)	51.6 ± 7.0	54.2 ± 8.8	51.2 ± 7.8	55.8 ± 8.5	54.4 ± 9.4
Disease duration (years)	—	5.0 (2-12)	3.5 (2-13)	7 (2.5-12.5)	4 (1.5-10)
RF (kU/l)^a^	—	65.1 (34.8-187)	63.1 (37.9-146.8)	62.1 (29.0-150)	93.5 (34.6-259)
RF-positive (%)^a^	—	62 (87.3)	15 (93.8)	24 (85.7)	23 (85.2)
Anti-CCP (U/ml)^b^	—	94.1 (31.7-532.3)	87.1 (22.4-536.1)	91.4 (49.2-501)	100.9 (34.8-574)
Anti-CCP-positive (%)^b^	—	67 (89.3)	16 (88.9)	26 (89.7)	25 (89.3)
CRP (mg/l)^C^	—	3.0 (0.9-9.9)	1.3 (0.8-3.6)	2.4 (0.9-9.3)	5.1 (1.2-17.3)
CRP positive (%)^C^	—	20 (28.6)	1 (6.7)	8 (27.6)	11 (42.3)
ESR (mm/h)	—	27 (18-42)	19 (11-26)	33 (23-46)	38 (21.5-51.5)
DAS28-ESR	—	4.4 ± 1.6	2.6 ± 0.4	4.0 ± 1.0	5.9 ± 0.9
Medicine use	—				
NSAIDs	—	16 (20.8)	2 (10.5)	8 (27.6)	6 (20.7)
DMARDs	—	57 (74.0)	17 (89.5)	20 (69.0)	20 (69.0)
Methotrexate	—	47 (61.0)	15 (78.9)	15 (51.7)	17 (58.6)
Leflunomide	—	32 (41.6)	10 (52.6)	11 (37.9)	11 (37.9)
Sulfasalazine	—	8 (10.4)	2 (10.5)	5 (17.2)	1 (3.4)
Prednisolone	—	12 (15.6)	2 (10.5)	3 (10.3)	7 (24.1)
Chinese medicine	—	16 (20.8)	4 (21.1)	7 (24.1)	7 (24.1)
No treatment	—	7 (9.1)	1 (5.3)	2 (6.9)	4 (13.8)
Other^d^	—	4 (5.2)	1 (5.3)	2 (6.9)	1 (3.4)
Unknown^e^	—	4 (5.2)	0 (0)	1 (3.4)	3 (10.3)

The data are expressed as *n* (%), mean ± standard deviation (SD), or median (interquartile range, 25th-75th). DAS28-ESR: Disease Activity Score of 28 joints using ESR; NSAIDs: nonsteroidal anti-inflammatory drugs; DMARDs: disease-modifying antirheumatic drugs. ^a^RF data were lacking in 6 subjects. Sixty-two out of 71 RA subjects were RF-positive. ^b^Anti-CCP data were lacking in 2 subjects. Sixty-seven out of 75 RA subjects were anti-CCP-positive. ^c^CRP data were lacking in 7 subjects. Twenty out of 70 RA subjects were CRP-positive. ^d^The patients defined as “other” included the following: those who were on their first visit to our hospital, were not on regular medication (one patient), and were taking other medicines that were not related to RA therapy (three patients). ^e^The patients defined as “unknown” included the following: those who were on their first visit to our hospital, could not tell which medication to use (two patients), and were only told of a few of their medicines (two patients).
