# Supplementary material for: Increased Expression of TLR10 in B Cell Subsets Correlates with Disease Activity in Rheumatoid Arthritis
Source: Mediators Inflamm. 2018 Dec 27;2018:9372436. doi: 10.1155/2018/9372436 (PMC6327257; doi:10.1155/2018/9372436)
Supplement: Supplementary Materials — Table S1: serum concentration of cytokines in all participants. Figure S1: the correlation between %TLR10+ (a) and TLR10 MFI (b) in CD19+ B cells and IL-1β concentrations. Figure S2: the correlation between %TLR10+ (a) and TLR10 MFI (b) in CD19+CD27+ B cells and ESR. Figure S3: (a) the correlation between %TLR10+ in CD27+IgD− B cells and ESR. (b) The correlation between TLR10 MFI in CD27+IgD+ B cells and ESR. [file 9372436.f1.pdf]

Table 1. Serum concentration of cytokines in all participants

Group	N	IL-IRα(pg/ml)	IL-Iβ(pg/ml)	IL-10(pg/ml)
Healthy control	30	678.91±131.18	12.85±1.80	1.18±0.16
All RA group	77	582.27±86.98	16.00±2.00	9.63±3.66*
Low activity	19	414.67±210.63	12.41±3.63	2.08±0.71
Moderate activity	29	582.18±126.04	15.24±2.14	8.56±3.40*
High activity	29	693.36±137.30	19.10±4.16	15.65±9.07**

Values described are the mean ± SE

* P< 0.05, ** P<0.01 versus healthy controls

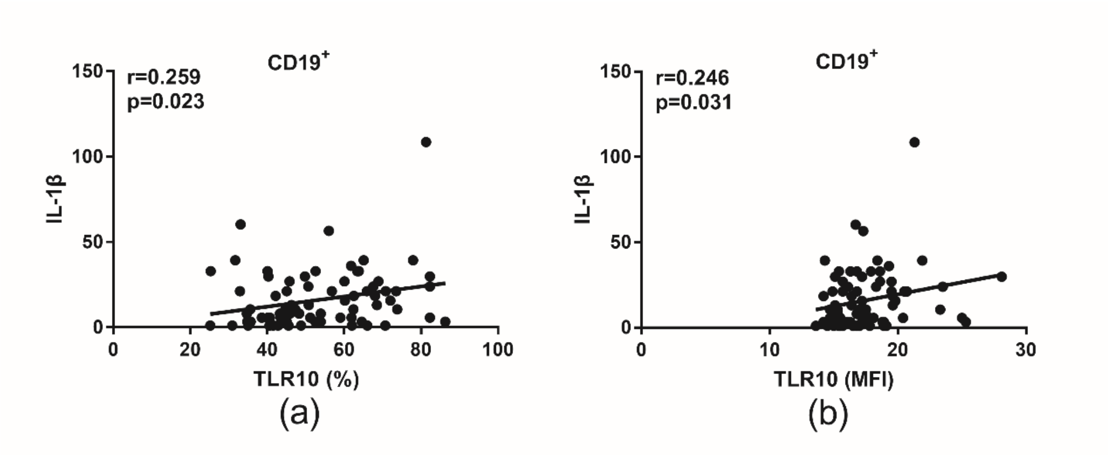

Figure 1: The correlation between %TLR10+ (a), TLR10 MFI (b) in CD19+ B cells and IL-1β concentrations.

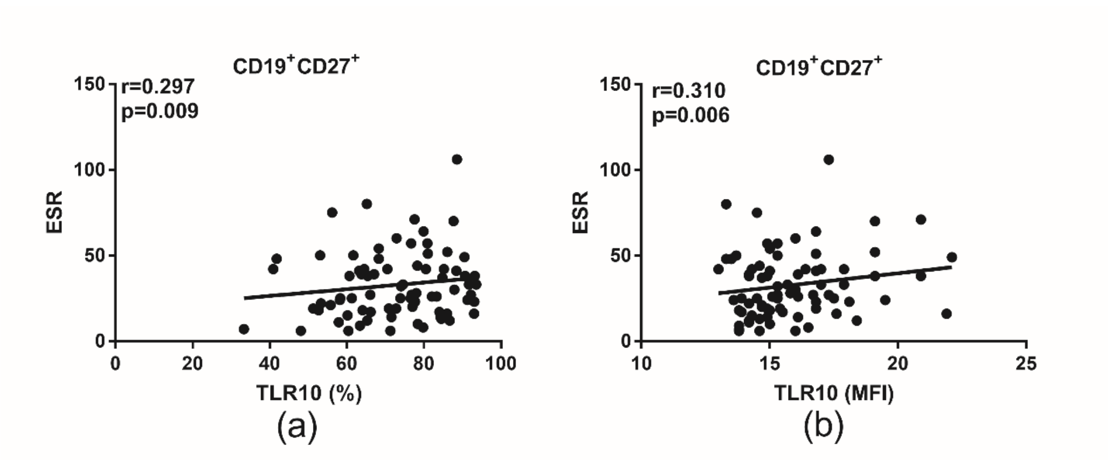

Figure 2: The correlation between %TLR10+ (a), TLR10 MFI (b) in CD19+CD27+ B cells and ESR.

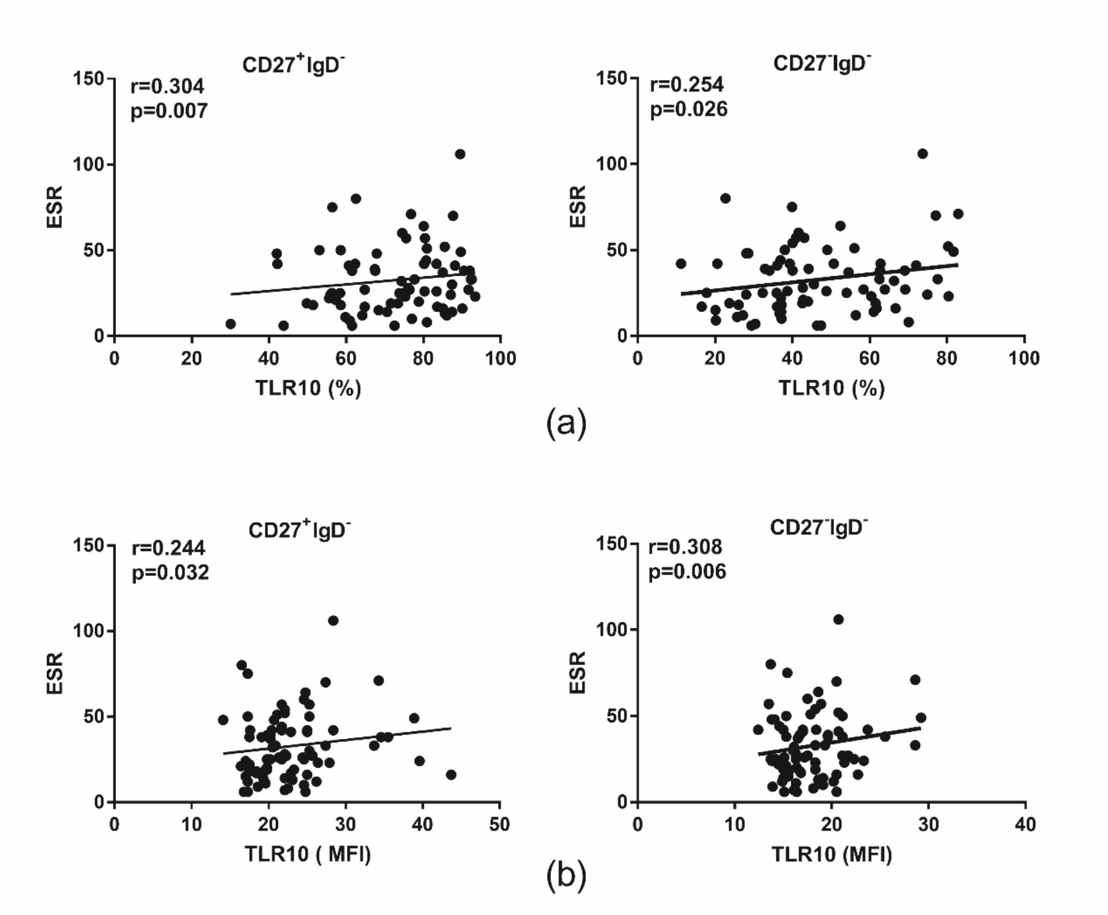

Figure 3: (a)The correlation between %TLR10+ in CD27+ IgD- B cells and ESR. (b) The correlation between TLR10 MFI in CD27+ IgD+ B cells and ESR.
